# Net benefit of surveillance varies by hepatocellular carcinoma risk in patients with cirrhosis

**DOI:** 10.1016/j.jhepr.2026.101831

**Published:** 2026-03-25

**Authors:** Akash Patel, Elena Gavrila, Sruthi Yekkaluri, Yujin Hoshida, Ruben Hernaez, Amit G. Singal

**Affiliations:** 1 Department of Internal Medicine, UT Southwestern Medical Center, Dallas, TX, USA; 2 Department of Medicine, Baylor College of Medicine, Houston, TX, USA; 3 Department of Medicine, Michael E. DeBakey Veterans Affairs Medical Center, Houston, TX, USA

**Keywords:** Liver cancer, Cirrhosis, Risk stratification, Precision screening, Net benefit

## Abstract

**Background:**

The risk of hepatocellular carcinoma (HCC) and net benefit of surveillance can differ between patients with cirrhosis. We compared the benefits and harms of HCC surveillance across risk strata in patients with cirrhosis.

**Methods:**

We leveraged a cohort of patients with cirrhosis from a pragmatic randomized controlled trial of HCC surveillance outreach enrolled between March 2018 and April 2021. Patients were stratified into low-, intermediate-, and high-risk categories based on published thresholds for three validated clinical risk scores. We calculated HCC incidence rates and moderate-to-severe physical harms for each risk stratum.

**Results:**

Of 2,142 patients with cirrhosis followed for a median of 36 months, 84 developed HCC and 90 (4.2%) experienced moderate-to-severe physical harm. The risk scores each achieved moderate discrimination for the prediction of HCC, with c-statistics ranging from 0.60 to 0.68. HCC incidence significantly increased from low- to high-risk categories across all clinical risk scores, whereas surveillance-related harms were similar across all risk strata. The net benefit (weighted benefit *vs.* harm assessment) was highest for high-risk stratum (range 2.8–3.9) and lowest for low-risk stratum (range from -0.3 to 0.1), indicating the greatest net benefit for high-risk patients and less clear benefit in low-risk patients. Despite variation in the overall value of surveillance across risk categories, adherence to surveillance was low (median proportion time covered by imaging: 32.3%) and not proportional to HCC risk.

**Conclusions:**

The net benefit of HCC surveillance significantly differs by HCC risk category in patients with cirrhosis, providing an impetus for risk-stratified surveillance approaches.

**Impact and implications:**

Surveillance is recommended in all patients with cirrhosis, although its net benefit can differ between patients based on risk of HCC. Using three validated clinical risk scores in 2,142 patients with cirrhosis followed for a median of 36 months, we found that HCC incidence significantly increased from low- to high-risk categories, whereas surveillance-related harms were similar across all risk strata. Therefore, the net benefit of HCC surveillance was greatest for high-risk individuals and lowest for low-risk stratum. These data provide an impetus to transition from one-size-fits-all surveillance to a risk-stratified or precision approach.

**Clinical Trials registration:**

NCT02582918.

## Highlights

• Over a median of 3 years, 84 of 2,142 cirrhosis patients developed HCC; 4.2% had moderate-to-severe harm.
• HCC risk scores showed moderate discrimination, with c-statistics of 0.60–0.68.
• HCC incidence rose across risk strata; whereas, surveillance harms were similar across strata.
• Net benefit was highest in high-risk patients and lowest or unclear in low-risk patients with cirrhosis.
• Surveillance adherence was low across all strata and not proportional to HCC risk.

## Graphical abstract

**Figure:**
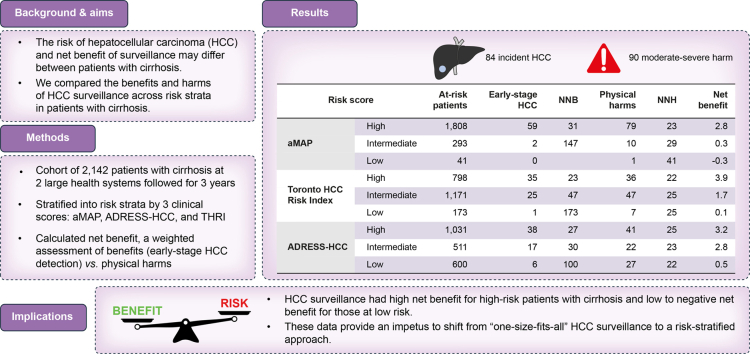

## Introduction

Hepatocellular carcinoma (HCC) is a leading cause of mortality among patients with cirrhosis.^1^ Prognosis for patients with HCC is associated with tumor stage, given that median survival exceeds 5 years with surgical therapies if detected at an early stage.^2^^,^^3^ Accordingly, at-risk patients are recommended to undergo semi-annual surveillance using abdominal ultrasound, with or without AFP measurements.^4^^,^^5^

HCC surveillance is supported by a large randomized controlled trial (RCT) in patients with chronic HBV infection and studies among patients with cirrhosis showing associations with early tumor detection and improved survival.^6^^,^^7^ However, benefits must be weighed against screening-related physical, financial, and psychological harms. Physical harms, including diagnostic evaluations for false positive or indeterminate results, are common but mostly mild in severity.^8^ However, patients with false positive results can also experience psychological and financial harms.^9^^,^^10^ A Delphi panel of experts emphasized the importance of evaluating both benefits and harms when considering the overall value of screening programs.^11^

Although HCC surveillance is cost-effective in patients with cirrhosis given an annual incidence exceeding 1%,^12^ HCC risk varies widely between patients. Several risk stratification tools have been developed using readily available demographic and clinical factors to identify patients at higher versus lower risk of developing HCC.[13], [14], [15], [16], [17] Although few have been sufficiently validated for routine use in clinical practice, several demonstrated good discrimination in HCC risk in internal validation cohorts.^18^ Most evaluations have focused on benefits of tailoring surveillance strategies to increase early-stage HCC detection, with fewer data looking at variation in harms across risk strata.^19^ Understanding the trade-off between surveillance benefits and harms across risk strata is important to inform the net benefit of risk-stratified approaches to HCC surveillance. Indeed, the US Preventive Services Task Force synthesizes the balance of benefits and harms to determine net benefit when deciding about recommendations.^20^ Herein, we examined the benefits and harms of HCC surveillance, across risk strata using several validated scores, in a multicenter cohort of patients with cirrhosis undergoing surveillance.

## Patients and methods

### Study population

As previously described, we performed a pragmatic RCT of mailed outreach for HCC surveillance among patients with Child-Pugh A or B cirrhosis from March 2018 to April 2021.^21^^,^^22^ The study was conducted at three health systems: UT Southwestern Medical Center, an academic tertiary care referral center; Parkland Health, an integrated safety-net health system; and the Michael E. DeBakey Veterans Affairs (VA) Medical Center. Patients were required to have at least one outpatient clinic visit in the year before randomization.

In brief, patients were randomly assigned in a 1:1 ratio to receive mailed outreach invitations for HCC surveillance or usual care with visit-based surveillance. Visit-based surveillance comprised providers ordering ultrasound, with or without AFP, when the patient was seen in clinic per usual care. For patients with normal ultrasound results, repeat surveillance was typically conducted every 6 months. If either surveillance result was abnormal (*i.e.* mass ≥1 cm on ultrasound or elevated AFP), the patient was referred for diagnostic multi-phase computed tomography (CT) or magnetic resonance imaging (MRI).

We conducted a *post hoc* retrospective analysis among patients followed at UT Southwestern or Parkland Health. Patients from Michael E DeBakey VA Medical Center were not included given a lack of available data to calculate clinical risk models. Patients with Child-Pugh C cirrhosis, uncontrolled hepatic encephalopathy, history of HCC, or history of liver transplantation were excluded. The study was approved by the Institutional Review Board of the UT Southwestern Medical Center (STU 062015-054).

### Clinical risk stratification scores

Demographic and clinical data, including age, sex, race and ethnicity, liver disease etiology, and liver disease severity, were collected for all patients at baseline. Liver disease etiology was classified as HCV (viremic *vs.* post-sustained virological response [SVR]), HBV, alcohol-associated liver disease (ALD), metabolic-dysfunction associated steatotic liver disease (MASLD), and other, with a hierarchical algorithm used in patients with multiple etiologies: HCV > HBV > ALD > other > MASLD. Liver disease severity was assessed by Child-Pugh score, with the severity of ascites and hepatic encephalopathy classified as none, mild or controlled, or severe and uncontrolled. We recorded baseline laboratory values, including platelet count, creatinine, albumin, bilirubin, and AFP. Scores were calculated using both complete patient data as well as imputed datasets using K-nearest neighbors (KNN) imputation for missing values.

Patients were stratified into low, intermediate, and high-risk categories based on validated, risk assessment tools. We used three scores (aMAP, Toronto HCC Risk Index, and ADRESS-HCC[15], [16], [17]) that are validated in patients of all cirrhosis etiologies (Table S1). These risk stratification models were selected given their inclusion of readily available clinical factors, whereas other models could not be calculated in this cohort. Cut-off values for risk categories (low, medium, and high) were based on published thresholds.

### Outcomes

We examined proportion time covered (PTC) by surveillance for the cohort overall, as well as across risk strata for each clinical risk score. As previously described,^23^ PTC was calculated by dividing the number of covered months (based on surveillance results) by the number of months of follow-up. Patients with a normal ultrasound or subcentimeter liver lesion were assigned 7 months of covered time,^24^ whereas those with a suspicious liver lesion ≥1 cm on ultrasound were assigned 3 months of coverage, consistent with proposed definitions for timely diagnostic evaluation.^25^ Patients with indeterminate liver nodules ≥1 cm on CT or MRI were assigned 3 months of coverage,^26^^,^^27^ and those with benign findings or lesions <1 cm were assigned 7 months of follow-up. Patients were censored at the time of primary liver cancer diagnosis, death, liver transplantation, or end of study follow-up (*i.e.* 3 years of follow-up).

We defined benefits of HCC surveillance as the proportion of patients with HCC detected at an early stage. A diagnosis of HCC was based on consistent histology or characteristic imaging appearance (*i.e.* presence of arterial phase hyperenhancement, delayed washout, and/or capsule appearance).^28^ We also captured diagnoses of intrahepatic cholangiocarcinoma (CCA), which was defined by histology or LR-M radiographic appearance concerning for CCA when a biopsy was not possible. Early-stage liver cancer was defined using the Barcelona Clinic Liver Cancer (BCLC) staging system for HCC and TNM staging system for CCA.^3^

Surveillance-related harms were measured by physical harms, defined as follow-up tests (*e.g.* CT, MRI, or liver biopsy) performed for positive or indeterminate surveillance results, as per an established taxonomy used across cancer screening programs.^29^ Harms were classified as mild *vs.* moderate-to-severe, with mild defined as receipt of a single diagnostic CT scan or MRI and moderate-to-severe harms defined as multiple diagnostic imaging studies or any invasive evaluation including biopsy.

### Statistical analyses

We evaluated the discriminatory ability for each of the clinical risk scores using AUROC analysis. Discrimination was assessed using Harrell’s c-index, a measure of the ability of each model to correctly rank patients by their predicted risk of developing HCC. Higher c-index values indicate better discrimination, with values of 0.5–0.7 considered moderate discrimination and values >0.7 good discrimination. We then examined PTC by surveillance across each of the risk groups. We calculated incidence rates of HCC for each risk strata (low-, medium-, and high-risk), with death and liver transplantation as competing outcomes, as well as the proportion of patients in each risk strata whose disease was detected at an early stage. We conducted a sensitivity analysis including intrahepatic CCA given the potential for incidental detection on surveillance imaging. We next calculated incidence rates of physical harms in each risk strata. For both analyses, patients were censored at 3 years (*i.e.* end of the study period). We used Fine–Gray regression analysis to evaluate for differences in incidence rates of benefits and harms between risk groups.

We calculated the number needed to benefit (NNB), based on early-stage HCC detection, and number needed to harm (NNH) according to risk strata. NNB and NNH were defined as 1/(difference in absolute risk). We also calculated net benefit, defined as:

[1] [(true positives/n)–[(false positives/n)x(p/1-p)]]x100, [1]

where n was the total sample size and *p* was defined as the weight for harms *vs.* benefits.^30^ Although there is no set weight for HCC surveillance, we used a weight of *p* = 0.1 (*i.e.* benefits given 10 times greater weight), given the greater clinical significance of early HCC detection compared with moderate-to-severe physical harms.^11^ Higher values indicated greater net benefit. Statistical significance was defined as *p* <0.05. All analyses were conducted using SAS 9.4 (SAS Institute Inc., Cary, NC, USA).

## Results

### Patient characteristics

Characteristics of the 2,142 eligible patients are detailed in Table 1. The median age of patients was 59.3 years and 58.1% were men. The cohort was diverse regarding race and ethnicity (30.3% non-Hispanic White, 27.4% Black, and 38.6% Hispanic) and liver disease etiology (22.6% with viremic HCV, 19.9% with post-SVR HCV, 23.8% with ALD, and 20.3% with MASLD). The median Child-Pugh score was 6, and 54.7% had Child-Pugh A cirrhosis.

**Table 1:** Patient characteristics.

Characteristics	Patients (N = 2,142)
Age (years)∗	59.3 (53.6–65.0)
Male sex (%)	1,244 (58.1)
Race/ethnicity (%)	
Non-Hispanic White	648 (30.3)
Hispanic White	827 (38.6)
Non-Hispanic Black	587 (27.4)
Other/unknown	80 (3.7)
Etiology of liver disease (%)	
Viremic HCV	484 (22.6)
Post-SVR HCV	427 (19.9)
Alcohol related	510 (23.8)
Metabolic dysfunction-associated steatohepatitis	435 (20.3)
HBV	78 (3.6)
Other	208 (9.7)
Presence of diabetes	814 (38)
Presence of ascites (%)	812 (37.9)
Presence of hepatic encephalopathy (%)	404 (18.9)
Child-Pugh score	6 (5–7)
Albumin (g/dl)∗^,^†	3.9 (3.4–4.2)
Bilirubin (mg/dl)∗^,^†	0.8 (0.5–1.3)
Platelet count∗^,^†	138 (91–186)

∗ Continuous variables reported as median (P25–P75).

† Albumin was missing in 342 (16%), bilirubin was missing in 321 (15%), and platelet count was missing in 57 (2.7%) patients.

Over the median follow-up of 36 months, 98 patients (4.6%) developed primary liver cancer, including 84 HCC and 14 CCA. Over two-thirds (70.4%) had early-stage tumors, including 82.1% of those with HCC and 57.1% of those with CCA.

### Discriminatory ability of clinical risk scores for incident HCC

Each clinical risk score stratified patients into various risk strata, although the proportion of high-risk *vs.* intermediate- or low-risk patients varied (Table 2). For example, 84.4% of patients were classified as high-risk using aMAP compared with only 37.3% of patients using Toronto HCC Risk Index. Conversely, 28.0% of patients were classified as low-risk by ADRESS-HCC, compared with 8.1% using the Toronto HCC Risk Index.

**Table 2:** Net benefit of HCC surveillance across clinical risk scores.

Risk score		Number of at-risk patients	Number early-stage HCC	NNB∗	Physical harms	NNH∗	Net benefit†
aMAP	High	1,808	59	31	79	23	2.8
	Intermediate	293	2	147	10	29	0.3
	Low	41	0	—	1	41	-0.3
Toronto HCC Risk Index	High	798	35	23	36	22	3.9
	Intermediate	1,171	25	47	47	25	1.7
	Low	173	1	173	7	25	0.1
ADRESS-HCC	High	1,031	38	27	41	25	3.2
	Intermediate	511	17	30	22	23	2.8
	Low	600	6	100	27	22	0.5

HCC, hepatocellular carcinoma; NNB, number needed to benefit; NNH, number needed to harm.

∗ NNB and NNH were defined over a 3-year follow-up period.

† Net benefit was calculated as [(true positives/n)–[(false positives/n)×(p/1-*p*)]]×100, where *p* is defined as the weight for benefits *vs*. harms (*p* = 0.1).

AUROC analyses for each risk score to predict HCC are shown in Fig. S1. Each clinical risk score achieved moderate discrimination for prediction of HCC, with c-statistics ranging from 0.60 to 0.68. Similar results were observed using the complete case dataset (c-statistics range: 0.60–0.69) and for prediction of HCC at 24 months (c-statistics range: 0.59–0.70).

### Surveillance benefits by risk strata

**Fig. 1:**
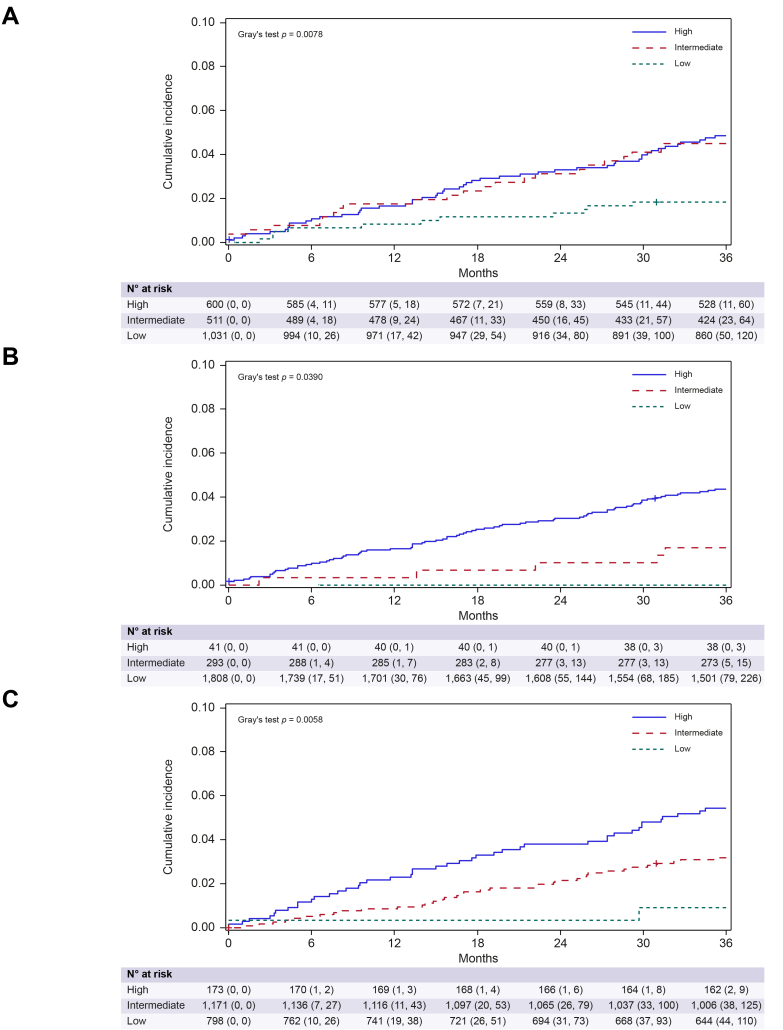
Incidence of HCC, stratified by risk category, per Fine-Gray analysis. HCC incidence increased across strata for clinical risk scores, including (A) ADRESS-HCC, (B) aMAP, and (C) Toronto HCC Risk Index. HCC, hepatocellular carcinoma.

HCC incidence significantly increased from low- to high-risk categories across clinical risk scores (Fig. 1). The 1- and 3-year cumulative incidences of HCC for low, intermediate, and high-risk groups for aMAPwere 0% and 0%, 0.3% and 1.7%, and 1.7 and 4.4%, respectively (*p* = 0.16 and *p* = 0.04); 0.8% and 1.8%, 1.8% and 4.5%, and 1.7% and 4.9% for ADRESS-HCC (*p* = 0.33 and *p* = 0.008); and 0.6% and 1.2%, 0.9% and 3.3%, and 2.4% and 5.5% for the Toronto HCC Risk Index (*p* = 0.02 and *p* = 0.006).

The proportions of patients with HCC who were detected by surveillance and those with early-stage detection did not significantly differ by risk strata. For example, 63.2% of patients with HCC in the Toronto HCC Risk Index intermediate-risk group and 70.5% in the high-risk group were detected by surveillance (*p* = 0.69). Similarly, 65.8% of patients in the intermediate-risk group and 79.6% of the high-risk group were detected at an early stage (*p* = 0.29).

In a sensitivity analysis including intrahepatic CCA, the 3-year cumulative incidences of PLC for low, intermediate, and high-risk groups were, respectively, 0%, 2.1%, and 5.1% for aMAP (*p* = 0.03); 1.8%, 4.9%, and 6% for ADRESS-HCC (*p* = 0.001); and 1.2%, 3.9%, and 6.3% for the Toronto HCC Risk Index (*p* = 0.004). The proportions of patients with PLC detected by surveillance and those with early-stage detection also did not significantly differ by risk strata. Using the Toronto HCC Risk Index as an example, intermediate and high-risk groups had no significant differences in surveillance detection (65.2% *vs.* 70.0%, *p* = 0.77) or early-stage detection (65.2% *vs.* 76.0%, *p* = 0.42).

### Surveillance harms by risk strata

**Fig. 2:**
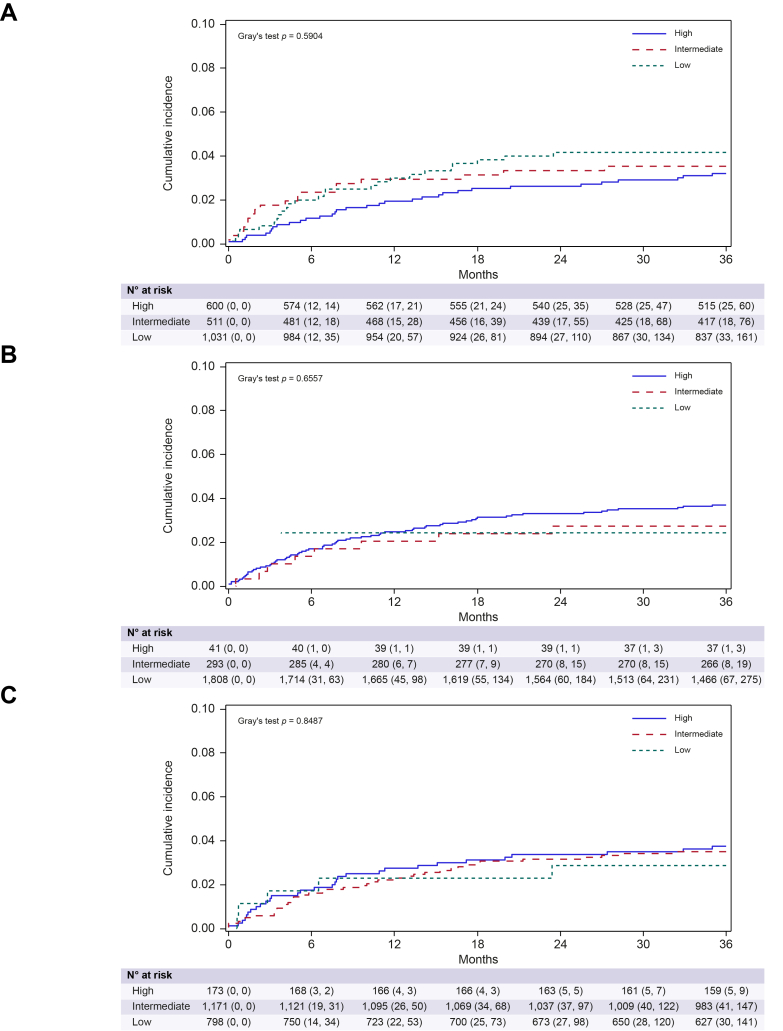
Incidence of moderate to severe physical harm, stratified by risk category, per Fine-Gray analysis. Moderate to severe harms did not significantly differ across risk strata for clinical risk scores in Fine–Gray regression analyses: (A) ADRESS-HCC (*p* = 0.17), (B) aMAP (*p* = 0.23), and (C) Toronto HCC Risk Index (*p* = 0.60).

Surveillance-related harms were observed in 202 (9.4%) patients, with 112 (5.2%) experiencing mild harm, 67 (3.1%) moderate harm, and 23 (1.1%) experiencing severe harm. There were no significant differences in physical harms across HCC risk categories. Three-year incidences of physical harms were 9.8%, 5.8%, and 8.7% across aMAP risk strata (*p* = 0.23); 10.2%, 7.8%, and 7.6% for ADRESS-HCC (*p* = 0.17); and 6.4%, 8.5%, and 8.7% for Toronto HCC Risk Index (*p* = 0.60). The proportion of moderate to severe physical harms also did not significantly differ from low- to high-risk categories, including 2.4%, 2.7%, and 3.7% for aMAP risk strata (*p* = 0.66), 4.2%, 3.5%, and 3.2%, respectively for ADRESS-HCC (*p* = 0.59), and 2.9%, 3.5%, and 3.8%, respectively for Toronto HCC Risk Index (*p* = 0.85) (Fig. 2).

### Net benefit of HCC surveillance by risk strata

The NNB for early-stage HCC detection was inversely correlated with risk strata across clinical risk scores (Table 2). Specifically, the NNB increased from 31 to 147 between the high and intermediate-risk strata for the aMAP score, from 23 to 47 between high and intermediate-risk strata for the Toronto HCC Risk Index, and from 27 to 100 between high and low-risk strata for ADRESS-HCC. Conversely, NNH did not significantly differ across risk strata for clinical risk scores, ranging from 22 to 41. Accordingly, the NNB-to-NNH ratios suggested the greatest value for high-risk strata. Similar results were seen for net benefit, with higher values for the high-risk strata compared with intermediate and low-risk strata. Indeed, negative values for net benefit were observed for the low-risk group using aMAP over a range of benefit-to-harms weights (Table S2).

Results were limited by small numbers in subgroup analyses among those with viremic HCV and post-SVR HCV; however, overall trends appeared consistent with the primary analysis (Table S3). Between the high and intermediate Toronto HCC Risk Index groups, NNB increased from 25 to 148 in those with viremic HCV and from 13 to 24 in those with post-SVR HCV infection, whereas NNH were similar. Net benefit decreased from 3.7 to 0.4 in patients with viremic HCV and from 7 to 4 in patients with post-SVR. Results were also consistent when stratified by site (Table S4) and sex (Table S5), although robust conclusions were limited by small sample sizes in subgroups.

Results were similar in a complete case analysis restricted to patients without missing data (Table S6). The NNB increased from 29 to 121 for aMAP risk strata, 21 to 134 for the Toronto HCC Risk Index, and 25 to 123 for ADRESS-HCC. Conversely, NNH did not significantly differ across risk strata for clinical risk scores, ranging from 19 to 34. The NNB-to-NNH ratios and net benefit values continued to suggest the greatest value for high-risk strata, including negative values for net benefit in the low-risk aMAP group.

### Adherence to HCC surveillance

**Fig. 3:**
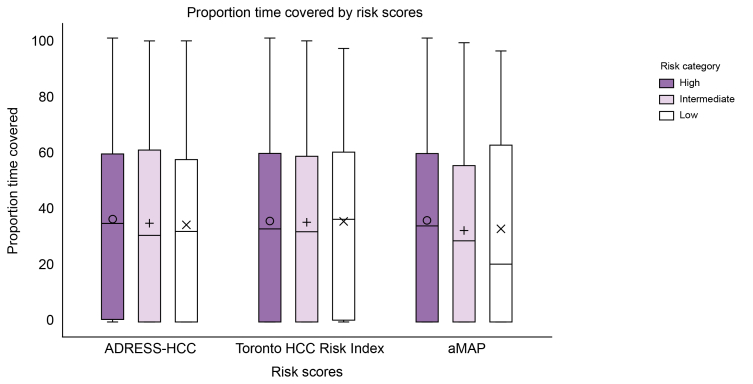
Proportion time covered by surveillance, stratified by risk category. Proportion time covered by surveillance did not significantly differ across risk strata for clinical risk scores: (A) ADRESS-HCC, (B) aMAP, and (C) Toronto HCC Risk Index.

The median PTC by imaging was 32.3% (P25–75: 0–58.5%) in the entire cohort. Although there were differences in the PTC by surveillance across risk strata, it was not proportion to patients’ HCC risk (Fig. 3). For example, median PTC was 36.2% (P25–75: 0.64–59.8%) in the low-risk group, 31.8% (P25–75: 0–58.4%) in intermediate-risk group, and 32.8% (P25-75: 0–59.4%) in the high-risk group as per the Toronto HCC Risk Index.

## Discussion

Our study adds to the literature evaluating a risk-stratified approach to HCC surveillance in patients with cirrhosis. Although HCC incidence increased across HCC risk strata for each of the clinical risk scores, physical harms did not appear to differ across risk strata. Therefore, the net benefit of HCC surveillance was greatest for high-risk patients and lowest for low-risk categories. Despite variation in net benefit of surveillance across risk categories, adherence to surveillance was low across all groups.

Understanding the benefits and harms of cancer screening is important for providing high-value care.^31^ Although several cancer screening programs demonstrated improvements in early-stage disease detection and, occasionally, mortality, they expose patients to unintended psychosocial, physical, and financial harms. A risk-stratified approach to cancer screening could aid optimization of the benefit-to-harm profile of programs. Current evidence supporting these strategies comes from simulated trials, suggesting improvements in outcomes, such as quality-adjusted life years or healthcare costs.[32], [33], [34] However, fewer empirical data support a transition to precision screening. A small retrospective study evaluating a risk-stratified approach for HCC surveillance similarly found that low-risk patients had a higher NNB than NNH, suggesting lower value of surveillance in this group.^19^ Our findings in a larger sample of patients reinforce the higher benefit-to-harm ratio in high-risk than in low-risk patients.

Consistent with previous research,^35^ we noted that surveillance adherence, as measured by proportion time covered by imaging, was low across risk categories. Previous studies have shown that surveillance underuse is related to both patient and provider barriers, underscoring a need for multilevel interventions to promote routine surveillance.^22^^,^^36^^,^^37^ Whereas most studies have evaluated interventions across all patients with cirrhosis, independent of HCC risk, it might be cost-effective for health systems to target interventions to the highest risk groups.^38^ Conversely, patients at low-risk might instead benefit from shared decision-making after discussion of potential benefits and harms.^11^ A previous survey study of providers reported willingness to adopt risk-stratified surveillance approaches, although providers were more likely to intensify surveillance in high-risk individuals than de-intensity in low-risk individuals.^39^

Several risk stratification tools have been developed to improve HCC surveillance among patients with cirrhosis. Here, we utilized the aMAP, Toronto HCC Risk Index, and ADRESS-HCC, which are validated in various populations, because they used readily available clinical infomation.[13], [14], [15], [16], [17] Each risk score stratifies patients, with 36-month cumulative incidences of HCC of 0–1.8%, 1.7–4.5%, and 4.4–5.5% for low, intermediate and high-risk patients, respectively. Notably, no patients categorized as low-risk by the aMAP or the Toronto HCC Risk Index developed HCC, underscoring the potential for scores with high negative predictive values to identify patients in whom surveillance is of low value and could be avoided. However, each score only had moderate discrimination, highlighting a need for improvement in accuracy. It is possible that more nuanced risk scores, incorporating genetics, or other biomarkers might facilitate more accurate risk stratification.[40], [41], [42], [43] Further research is warranted to determine which combination of tools would be best to use before a risk-stratified surveillance approach for HCC can be adopted.

Our findings should be interpreted within study limitations. Some patients were missing relevant data points needed to calculate their risk scores. Although KNN imputation methods have demonstrated accuracy, these do not serve as an exact proxy.^44^^,^^45^ In addition, although surveillance utilization was similar to previous estimates, poor utilization might have impacted our findings for both surveillance-related harms and benefits. Third, we only had access to information on physical-related harms, which fails to characterize the full spectrum of surveillance-related harms, namely psychological or financial.^9^^,^^10^ Similarly, early-stage detection is only one step in the cancer care continuum and downstream failures, including treatment delays and underuse of curative therapies, can impact survival.^25^^,^^46^^,^^47^ Finally, our study evaluated surveillance using ultrasound with or without AFP, and future efforts would need to see if similar results incorporating clinical risk scores would be true for emerging imaging and blood-based strategies.[48], [49], [50]

In summary, our study highlights the potential of using validated risk stratification tools to refine HCC surveillance strategies for patients with cirrhosis. Accurately categorizing patients based on their risk of developing HCC can inform discussions about the benefit-to-harm ratio of HCC surveillance. Continued efforts to improve risk stratification are important to facilitating a transition from a one-size-fits-all approach to a precision surveillance strategy.

## Abbreviations

ALD, alcohol-associated liver disease; BCLC, Barcelona Clinic Liver Cancer; CCA, intrahepatic cholangiocarcinoma; CT, computed tomography; HCC, hepatocellular carcinoma; KNN, K-nearest neighbors; MASLD, metabolic-dysfunction associated steatotic liver disease; MRI, magnetic resonance imaging; NNB, number needed to benefit; NNH, number needed to harm; PTC, proportion time covered; RCT, randomized controlled trial; SVR, sustained virological response.

## Authors’ contributions

Study concept and design, drafting of the manuscript, funding acquisition, study supervision: AGS.Acquisition of data: EG, AP, RH, AGS.Analysis of data: SY.Interpretation of the data, critical revision of the manuscript for intellectual content: all authors.Guarantors of the article and take responsibility for the integrity of the research: AGS, RH.

## Data availability

Data supporting the findings of this study are available within the article and its supplementary materials. Other study materials and data related to the study are available from the corresponding author, upon reasonable request.

## Financial support

AGS received support from the 10.13039/100000054National Cancer Institute (U01 CA283935, P50 CA295495, R01 CA222900, R01 CA212008, and U01 CA271887 and CPRIT RP200554). YH is supported by 10.13039/100000002NIH (R01CA255621, R01CA282178, R01CA233794, U01CA288375, U01CA226052, and U01CA283935), 10.13039/501100000780European Commission (ERC-AdG-2020-101021417), and 10.13039/100004917CPRIT (RR180016). RH is a core faculty and also supported, in part, by the Center for Innovations in Quality, Effectiveness and Safety (CIN 13-413). The content is solely the responsibility of the authors and does not necessarily represent the official views of the NIH, Cancer Prevention Research Institute of Texas, or the US Government.

## Conflicts of interest

AGS has served as a consultant or on advisory boards for Genentech, AstraZeneca, Eisai, Exelixis, Bayer, Merck, Elevar, Boston Scientific, Sirtex, FujiFilm Medical Sciences, Exact Sciences, Hello Genomics Roche, Glycotest, Abbott, IMCare, Curve Biosciences, DELFI, and Universal Dx. The other authors have no relevant conflicts of interest to declare.

Please refer to the accompanying ICMJE disclosure forms for further details.
