# Supplementary material for: Net benefit of surveillance varies by hepatocellular carcinoma risk in patients with cirrhosis
Source: JHEP Rep. 2026 Mar 25;8(8):101831. doi: 10.1016/j.jhepr.2026.101831 (PMC13380099; doi:10.1016/j.jhepr.2026.101831)
Supplement: Multimedia component 1 [file mmc1.pdf]

Net benefit of surveillance varies by hepatocellular carcinoma risk in patients with cirrhosis

Akash Patel, Elena Gavrila, Sruthi Yekkaluri, Yujin Hoshida, Ruben Hernaez, Amit G. Singal

Table of contents

Table S1………………………………………………………………………………… Page 2

Table S2………………………………………………………………………………… Page 3

Table S3………………………………………………………………………………… Page 4

Table S4………………………………………………………………………………… Page 5

Table S5………………………………………………………………………………… Page 6

Table S6………………………………………………………………………………… Page 7

Fig. S1..………………………………………………………………………………… Page 8

Table S1. Clinical risk scores used for risk stratification

Risk Score	Components	Categories
aMAP	Age, sex, albumin-bilirubin (ALBI) score, platelets	Low: ≤ 50<br>Intermediate: (>50 – < 60)<br>High: ≥ 60
Toronto HCC Risk Index	Age, gender, liver disease etiology, platelets	Low: < 120<br>Intermediate: (120 – 240)<br>High: > 240
ADRESS-HCC	Age, diabetes, race, liver disease etiology, sex, liver disease severity.	Low: < 4.71<br>Intermediate: (4.71 – 5.24)<br>High: >5.24

Table S2. Net benefit* of HCC surveillance across a range of benefit-to-harm weights

Risk Score		Number of patients	p=0.05	p=0.1**	p=0.2	p=0.3	p=0.4	p=0.5
aMAP	High	1808	3.0	2.8	2.2	1.4	0.3	-1.1
	Intermediate	293	0.5	0.3	-0.2	-0.8	-1.6	-2.7
	Low	41	-0.1	-0.3	-0.6	-1.0	-1.6	-2.4
Toronto HCC Risk Index	High	798	4.1	3.9	3.3	2.4	1.4	-0.1
	Intermediate	1171	1.9	1.7	1.1	0.4	-0.6	-1.9
	Low	173	0.4	0.1	-0.4	-1.2	-2.1	-3.5
ADRESS-HCC	High	1031	3.5	3.2	2.7	2.0	1.0	-0.3
	Intermediate	511	3.1	2.8	2.3	1.5	0.4	-1.0
	Low	600	0.8	0.5	-0.1	-0.9	-2.0	-3.5

* Net benefit was calculated as [(true positives/N) – [(false positives/N) * (p/1-p)]] * 100, where p is defined as the weight for benefits vs. harms

** Weight used in the primary analysis

Table S3. Net benefit of HCC surveillance across clinical risk scores, stratified by viremic vs. post-SVR hepatitis C subgroups

Risk Score			Number of patients	Number early-stage HCC	NNB*	Physical harms	NNH*	Net benefit**
aMAP	High	Viremic	427	13	33	10	43	2.8
		Post SVR	365	17	21	14	26	4.2
	Intermediate	Viremic	51	1	51	1	51	1.7
		Post SVR	58	---	---	2	29	-0.4
	Low	Viremic	6	---	---	---	---	---
		Post SVR	4	---	---	---	---	---
Toronto HCC Risk Index	High	Viremic	331	13	25	7	47	3.7
		Post SVR	38	3	13	3	13	7.0
	Intermediate	Viremic	148	1	148	4	37	0.4
		Post SVR	317	13	24	2	159	4.0
	Low	Viremic	5	---	---	---	---	---
		Post SVR	72	1	72	---	---	1.4
ADDRESS HCC	High	Viremic	401	14	29	11	36	3.2
		Post SVR	360	17	21	11	33	4.4
	Intermediate	Viremic	67	---	---	---	---	---
		Post SVR	56	---	---	4	14	-0.8
	Low	Viremic	16	---	---	---	---	---
		Post SVR	11	---	---	1	---	-1.0

HCC – hepatocellular carcinoma; NNB – number needed to benefit; NNH – number needed to harm

* Number needed to benefit and number needed to harm were defined over a 3-year follow-up period

** Net benefit was calculated as [(true positives/N) – [(false positives/N) * (p/1-p)]] * 100, where p is defined as the weight for benefits vs. harms (p=0.1).

Table S4. Net benefit of HCC surveillance across clinical risk scores, stratified by site

Risk Score			Number of patients	Number early-stage HCC	NNB*	Physical harms	NNH*	Net benefit**
aMAP	High	UTSW	359	15	24	18	20	3.6
		Parkland	1449	44	33	61	24	2.6
	Intermediate	UTSW	58	1	58	3	19	1.1
		Parkland	235	1	235	7	34	0.1
	Low	UTSW	10	---	---	---	---	---
		Parkland	31	1	31	1	31	2.9
Toronto HCC Risk Index	High	UTSW	140	10	14	3	47	6.9
		Parkland	658	25	26	33	20	3.2
	Intermediate	UTSW	244	6	41	17	14	1.7
		Parkland	927	19	49	30	31	1.7
	Low	UTSW	43	---	---	1	43	---
		Parkland	130	1	130	6	22	0.3
ADDRESS HCC	High	UTSW	140	6	23	4	35	4.0
		Parkland	891	32	28	37	24	3.1
	Intermediate	UTSW	115	5	23	4	29	4.0
		Parkland	396	12	33	18	22	2.5
	Low	UTSW	172	5	34	13	13	2.1
		Parkland	428	1	428	14	31	-0.1

HCC – hepatocellular carcinoma; NNB – number needed to benefit; NNH – number needed to harm

* Number needed to benefit and number needed to harm were defined over a 3-year follow-up period

** Net benefit was calculated as [(true positives/N) – [(false positives/N) * (p/1-p)]] * 100, where p is defined as the weight for benefits vs. harms (p=0.1).

Table S5. Net benefit of HCC surveillance across clinical risk scores, stratified by sex

Risk Score			Number of patients	Number early-stage HCC	NNB*	Physical harms	NNH*	Net benefit**
aMAP	High	Male	1147	44	26	52	22	3.3
		Female	661	15	44	27	24	1.8
	Intermediate	Male	88	---	---	0	---	---
		Female	205	2	103	10	21	0.4
	Low	Male	9	---	---	---	---	---
		Female	32	---	---	1	32	---
Toronto HCC Risk Index	High	Male	710	30	24	33	22	3.7
		Female	88	5	18	3	29	5.3
	Intermediate	Male	530	14	38	19	28	2.2
		Female	641	11	58	28	23	1.2
	Low	Male	4	---	---	---	---	---
		Female	169	1	169	7	24	0.1
ADDRESS HCC	High	Male	739	31	24	30	25	3.7
		Female	292	7	42	11	27	2.0
	Intermediate	Male	266	11	24	15	18	3.5
		Female	245	6	41	7	35	2.1
	Low	Male	239	2	120	7	34	0.5
		Female	361	4	90	20	18	0.5

HCC – hepatocellular carcinoma; NNB – number needed to benefit; NNH – number needed to harm

* Number needed to benefit and number needed to harm were defined over a 3-year follow-up period

** Net benefit was calculated as [(true positives/N) – [(false positives/N) * (p/1-p)]] * 100, where p is defined as the weight for benefits vs. harms (p=0.1).

Table S6. Net benefit of HCC surveillance across clinical risk scores in complete case analysis (i.e., patients without missing data for clinical risk scores)

**Risk Score**		**Number of patients**	**Number early-stage HCC**	**NNB***	**Physical harms**	**NNH***	**Net benefit****
aMAP	High	1492	51	29	69	22	2.9
	Intermediate	242	2	121	9	27	0.4
	Low	34	---	---	1	34	-0.3
Toronto HCC Risk Index	High	685	33	21	32	21	4.3
	Intermediate	949	19	50	40	24	1.5
	Low	134	1	134	7	19	0.2
ADRESS-HCC	High	844	34	25	37	23	3.5
	Intermediate	432	15	29	21	21	2.9
	Low	492	4	123	21	23	0.3

HCC – hepatocellular carcinoma; NNB – number needed to benefit; NNH – number needed to harm

* Number needed to benefit and number needed to harm were defined over a 3-year follow-up period

** Net benefit was calculated as [(true positives/N) – [(false positives/N) * (p/1-p)]] * 100, where p is defined as the weight for benefits vs. harms (p=0.1).

FIGURE LEGENDS

Figure S1. Receiver operating characteristic curves for discrimination of hepatocellular carcinoma (HCC)

Clinical risk scores achieved moderate discrimination for HCC at 3 years
