# Supplementary material for: Net benefit of surveillance varies by hepatocellular carcinoma risk in patients with cirrhosis
Source: JHEP Rep. 2026 Mar 25;8(8):101831. doi: 10.1016/j.jhepr.2026.101831 (PMC13380099; doi:10.1016/j.jhepr.2026.101831)
Supplement: Multimedia component 2 [file mmc2.docx]

**JHEP Reports**

**CTAT methods**

Tables for a “Complete, Transparent, Accurate and Timely account” (CTAT) are now mandatory for all revised submissions. The aim is to enhance the reproducibility of methods.

- Only include the parts relevant to your study
- Refer to the CTAT in the main text as ‘Supplementary CTAT Table’
- Do not add subheadings
- Add as many rows as needed to include all information
- Only include one item per row

**If the CTAT form is not relevant to your study, please outline the reasons why:**

|  |
| --- |

- 1. **Please provide the details of the corresponding methods author for the manuscript:**

| **Amit Singal, amit.singal@utsouthwestern.edu** |
| --- |

**2.0 Please confirm for randomised controlled trials all versions of the clinical protocol are included in the submission. These will be published online as supplementary information.**

| **N/A** |
| --- |
